# Eye-masks and earplugs compared to headband in nulliparas on increasing spontaneous vaginal delivery: a randomized trial

**DOI:** 10.1186/s12884-023-05685-4

**Published:** 2023-05-24

**Authors:** Jesrine Gek Shan Hong, Annamalai Vimaladevi, Noor Adeebah Razif, Siti Zawiah Omar, Peng Chiong Tan

**Affiliations:** 1 Department of Obstetrics and Gynecology, Faculty of Medicine, Universiti Malaya, Jalan Profesor Diraja Ungku Aziz, 50603 Kuala Lumpur, Malaysia; 2 Trinity College Dublin, University of Dublin, Dublin 2, College Green, D02 PN40 Ireland

**Keywords:** Eye-mask, Earplug; sleep, Pregnancy, Nullipara, Vaginal delivery, Cesarean delivery

## Abstract

**Background:**

A majority of pregnant women experience sleep disruption during pregnancy, especially in the third trimester. Lack of sleep is associated with preterm birth, prolonged labor and higher cesarean section rate. Six or less hours of night sleep in the last month of pregnancy is associated with a higher rate of caesarean births. Eye-masks and earplugs compared to headband improve night sleep by 30 or more minutes. We sought to evaluate eye-mask and earplugs compared to sham/placebo headbands on spontaneous vaginal delivery.

**Methods:**

This randomized trial was conducted from December 2019-June 2020. 234 nulliparas, 34–36 weeks’ gestation with self-reported night sleep < 6 h were randomized to eye-mask and earplugs or sham/placebo headband (both characterized as sleep aids) to be worn each night to delivery. After two weeks, interim outcome data of the average night sleep duration and the trial sleep related questionnaire was answered through the telephone.

**Results:**

Spontaneous vaginal delivery rates were 60/117(51.3%) vs. 52/117(44.4%) RR 1.15 95% CI 0.88–1.51 *P* = 0.30 for eye-mask and earplugs or headband respectively. At 2-weeks into the intervention period, the eye-mask and earplugs arm reported longer night sleep duration 7.0 ± 1.2 vs. 6.6 ± 1.5 h *P* = 0.04, expressed increased satisfaction with the allocated aid 7[6.0–8.0] vs. 6[5.0–7.5] *P* < 0.001, agreed they slept better 87/117(74.4%) vs. 48/117(41.0%) RR 1.81 95% CI 1.42–2.30 NNT_b_ 4 (2.2–4.7) *P* < 0.001 and higher compliance median[interquartile range] 5[3–7] vs. 4[ 2–5] times per week of sleep aid use *P* = 0.002.

**Conclusion:**

Eye-mask and earplugs use at home in late third trimester do not increase the spontaneous vaginal delivery rate even though self-reported night sleep duration, sleep quality, satisfaction and compliance with allocated sleep aid were significantly better than for sham/placebo headband.

**Trial registration**

This trial was registered with ISRCTN on June 11, 2019 with trial identification number: ISRCTN99834087.

**Supplementary Information:**

The online version contains supplementary material available at 10.1186/s12884-023-05685-4.

## Condensation

Eye-mask and earplugs use in late pregnancy amongst short sleepers do not significantly increase the spontaneous vaginal delivery rate.

## Background

A majority of pregnant women experience some form of sleep disruption during pregnancy [1–4]. The rate of sleep disturbances also increases across trimesters, ranging from 13% in the first trimester, 19% in the second, and 66% in the third [1, 5]. Lack of sleep in the third trimester has detrimental effect on pregnancy outcomes [1, 6]. Women who reported less than 6 h of sleep per night during the last month of pregnancy had a significantly longer mean duration of labor (29 h vs. ≤ 20 h) and a higher rate of cesarean births (< 6 h: 37%; 6–6.9 h: 34%; 7 + hours: 11%) [6]. Women who slept less than 7 h at night are at increased risk of developing gestational diabetes, gestational hypertension and preterm birth [7–9].

In an original trial report [10] of 56 women in late pregnancy done at our center, home-use of eye-masks and earplugs (EMEP) as sensory deprivation sleep aid and sham/placebo headband (HB) both significantly prolonged night sleep duration indicating an appreciable placebo effect of headbands as a purported sleep aid; a significantly higher proportion in EMEP arm prolonged their night sleep by at least 30 min (relative risk (RR) 2.3 95% CI 1.0–5.6) as per sleep actigraphy protocol and the point estimate for spontaneous vaginal delivery rates were 76.9% vs 57.7% (RR 1.2 95% CI 0.9–1.9), non-significantly higher for EMEP. EMEP improves sleep quality and sleep duration by 40–60% among patients nursed in intensive care unit [11]. In post-anesthesia care unit, EMEP reduces frequency of night awakenings and self-administered opioid use during the first night after surgery [12].

Nulliparas with their untested delivery status experienced higher cesarean and instrumental vaginal delivery rates compared to women with prior vaginal births [13]. Unplanned cesarean delivery [14, 15] and instrumental vaginal delivery [16, 17] are associated with adverse downstream outcomes [18]. Avoiding the index cesarean in nulliparous women is important to ensure a low risk obstetric future [19]. It is anticipated that nulliparas with short sleep will have higher rates of operative delivery.

We hypothesized that at home use of eye-masks and earplugs in their late third trimester may improve the spontaneous vaginal delivery rate among nulliparas. We performed a study on EMEP compared to sham HB as sleep aids to be used until delivery in nulliparas at 34–36 weeks predicated on increasing the spontaneous vaginal delivery rate as the primary outcome.

## Methods

This randomized controlled clinical intervention trial was approved by the Medical Ethics Committee of University Malaya Medical Center (date of approval 06/03/2019; reference number 201936–7199) and registered in ISRCTN registry on 11/06/2019 (registration number ISRCTN99834087 (https://doi.org/10.1186/ISRCTN99834087). The trial was conducted in accordance with the Declaration of Helsinki in University Malaya Medical Center with the first participant recruited on 16/12/2019 and the last on 16/06/2020.

### Participants

Nulliparas (no prior pregnancy beyond 20 weeks), aged ≥ 18 years, singleton pregnancy between 34 to 36 weeks gestation with self-reported night sleep of less than 6 h [10, 20] and access to the telephone were identified for recruitment in the antenatal clinic during their regular visits. In our center, all women booked to deliver in our hospital attend the antenatal clinic. As our center is a tertiary referral hospital, both low and high-risk pregnancies are seen in antenatal clinic. Based on an observational cohort study conducted in our center [21], the spontaneous vaginal birth rate was 70.3% and 20.6% deliveries were induced. Exclusion criteria were pre-existing sleep disorder (e.g. sleep apnea, insomnia), psychiatric disorder (e.g. depression, schizophrenia, bipolar mood disorder), underlying medical disorder that could affect sleep (e.g. lupus, heart disease, epilepsy, thyroid disorder), night shift workers or night care commitments (care takers of dependent family members), active smokers, current alcohol consumption, obesity (Class II and above; body mass index > 35 kg/m^2^), intrauterine death or fetus with gross anomaly. Our study also recruited only nulliparas to minimize potential bias from having to care for young children hence affecting sleep and to remove the risk of night call for attention by young dependents not being heeded.

Eligible women who attended antenatal clinic for regular follow-up were approached, assessed for eligibility, provided with the patient information sheet and verbally counselled with regard to trial participation by an investigator (co-author AV). Written consent was obtained from all participants.

### Randomization and interventions

Randomization to EMEP (intervention) or HB (sham/placebo) was through the strict sequential opening of lowest numbered available sealed and opaque envelope. Randomization sequences were prepared using a random number sequencer (random.org) in random blocks of 4 or 8 and within block by a co-author (PCT) who was not involved in recruitment.

Participants randomized to intervention were provided with eye-mask and earplugs were instructed to wear them when they go to bed at night until delivery. EMEP can be removed if the participant needed to mobilize during the night but to be re-worn on returning to bed to sleep. Participants randomized to HB were instructed to wear it circumferentially at the level of the brow when they go to bed to sleep at night. The sleep aids were to be removed upon wakening [10]. Both EMEP and HB were characterized as sleep aids to participants as specified to our institution ethics review board for this approach. We did not attempt to mask the interventions due to their obvious nature.

After a two-week interval, co-investigator (AV) made a phone call to the participants and asked four questions through an ad hoc sleep questionnaire to assess the sleep quality. Participants were asked to provide experience data on the two weeks since starting the allocated sleep aid intervention; 1) a 5-grade Likert scale response to “since using the sleep aid for the last one week, I have slept better”, 2) to rate using a 11-point visual numerical rating scale (VNRS rated from 0 to 10, higher score greater satisfaction) their “satisfaction with the use of allocated sleep aid” 3) to estimate average night sleep duration (hours) and 4) to give an estimate number of times use of their allocated sleep aid in a week. Standard maternity care was provided to all participants.

Sleep aids were purchased from an online store at a cost of less than USD 1 per set and a new set of allocated sleep aids were provided to each participant as allocated and reused nightly by them during the period of study until no longer fit for purpose. Labor and birth outcomes were retrieved from hospital records after participants’ delivery. All participants’ relevant demographic and clinical data were transcribed onto the Case Report Form.

### Outcome measures

Primary outcome was spontaneous vaginal delivery rate. Secondary outcomes include self-reported mean night sleep duration, maternal satisfaction with sleep aid and self-reported sleep quality as well as labor induction and indication, delivery blood loss, labor analgesia, mode of delivery, birthweight, umbilical cord arterial pH, Apgar scores, neonatal admission and indication. These labor and delivery outcomes were retrieved from the participants’ electronic medical record.

### Sample size calculation

On our primary outcome of spontaneous vaginal delivery rate, we used as pilot data the per protocol rates of 20/26 [76.9%] (EMEP) vs. 15/26 [57.7%] (HB) from Teo et al. [10]. Applying power of 80%, alpha of 0.05, 1 to 1 ratio across trial arms and the Chi-square test for analysis, 93 participants were required in each arm for a trial powered to these metrics. Assuming drop-out rate of 20%, rounded up to whole numbers, 117 (93/0.8) were needed in each arm. The total of participants planned was 234.

### Statistical analyses

Data were entered into a statistical software package SPSS (Version 23, IBM, SPSS Statistics). The student t test was used to analyze means with normal data distribution, the Mann–Whitney U test for non-normally distributed data or ordinal data, Chi-square test for categorical data (Fisher’s exact test if > 20% of cells evaluated have cell value < 5). Two-sided *P* values were reported and *P* < 0.05 was regarded as significant. We did not plan to perform statistical correction for multiple testing of secondary outcomes. Primary analysis was on intention-to-treat basis.

## Results

**Fig. 1:**
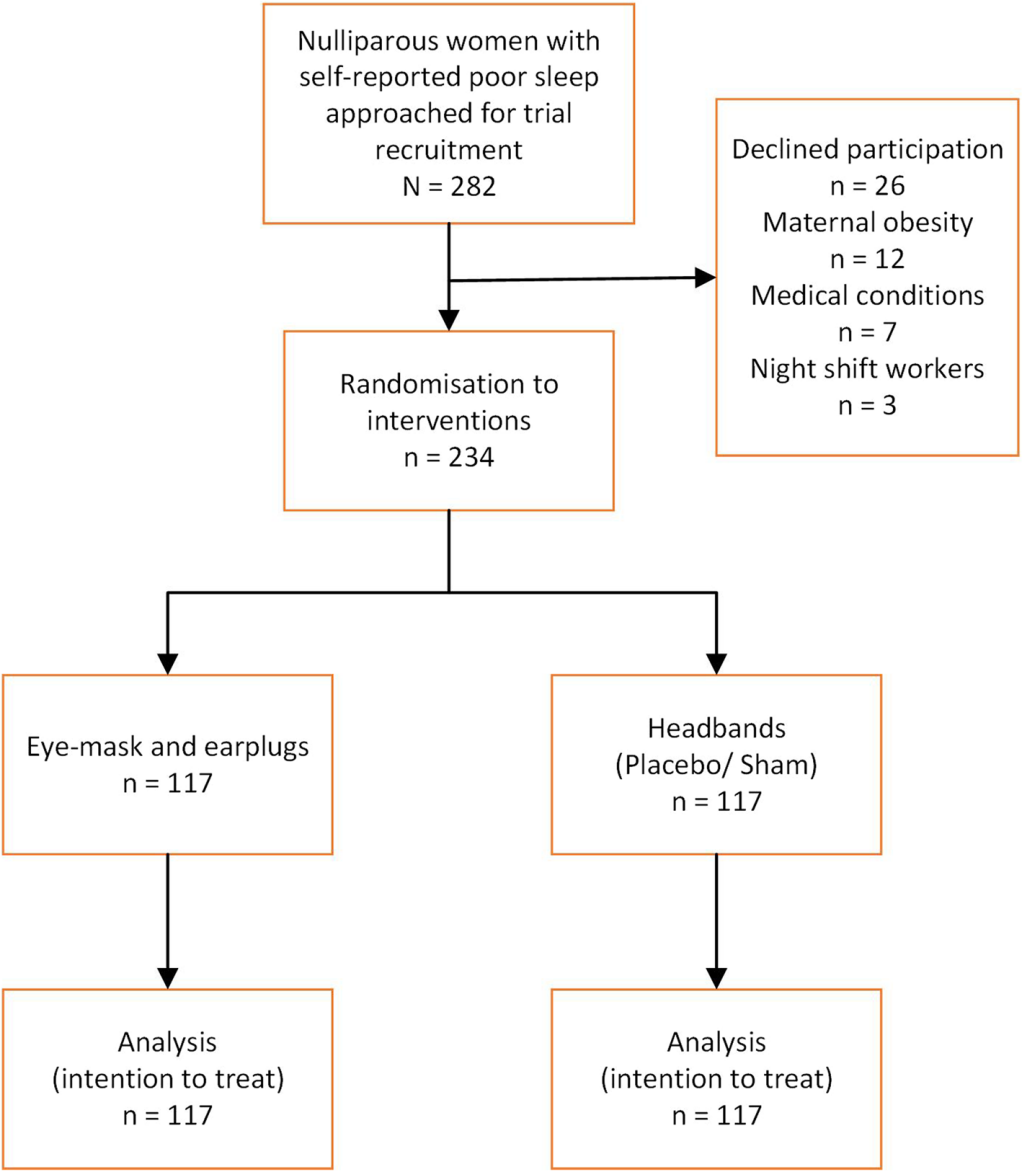
Recruitment flow chart of a randomized trial of eye-masks and earplugs compared with headbands in nulliparas

Figure 1 depicts the recruitment flow of participants through the study. Of the 282 potentially eligible women with self-reported sleep of less than 6 h approached, 256 agreed to participate; 22 did not fulfil other eligibility criteria (12 with maternal obesity > 35 kg/m^2^, seven with specified underlying medical conditions that could affect night sleep and three were night shift workers) which left with 234 to be randomized to EMEP (*n* = 117) and HB (*n* = 117). We included all participants for analysis based on the intention to treat principle. We stopped recruitment on reaching sample size target.

Table 1 shows the characteristics of the trial participants which were similar across trial arms. The gestational age at recruitment were 35.2 ± 1.2 weeks vs. 35.1 ± 1.1 weeks *P* = 0.79 for EMEP and HB arms respectively. All characteristics were similar across trial arms.

**Table 1:** Characteristics of nulliparous trial participants randomized to eye-mask and earplugs or headband

Characteristics	Eye-mask-earplugs *N* = 117	Headband *N* = 117	*P* value
Age (years)	30.2 ± 4.5	30.0 ± 4.7	0.70
Gestational age at recruitment (weeks)	35.2 ± 1.2	35.1 ± 1.1	0.79
Body mass index (kg/m2)	28.4 ± 4.2	27.9 ± 4.0	0.43
Ethnicity			
Malay	59 (50.4%)	75 (64.1%)	0.17
Chinese	32 (27.4%)	26 (22.2%)	
Indian	22 (18.8%)	14 (12.0%)	
Others^a^	4 (3.4%)	2 (1.7%)	
Occupation			
Paid employment	82 (70.1%)	76 (65.0%)	0.49
Homemakers or in training	35 (29.9%)	41 (35.0%)	
House type			
Semi-detached	12 (10.3%)	15 (12.8%)	0.83
Terrace	45 (38.5%)	44 (37.6%)	
Condominium/ flat	60 (51.3%)	58 (49.6%)	
Sharing bedroom	4 (3.4%)	2 (1.7%)	0.68^b^
Room ventilation			
Fan	43 (36.8%)	49 (41.9%)	0.70
Air-conditioned	62 (53.0%)	58 (49.6%)	
Fan & air-conditioned	12 (10.3%)	10 (8.5%)	
Type of bed			
Double	116 (99.1%)	116 (99.1%)	1.00^b^
Single	1 (0.9%)	1 (0.9%)	
Night light	20 (17.1%)	17 (14.5%)	0.72
Shift work (no night shift)	21 (17.9%)	32 (27.4%)	0.12
Gestational diabetes mellitus	35 (29.9%)	29 (24.8%)	0.38
Diet control	17 (48.6%)	18 (62.1%)	0.53
Metformin	14 (40.0%)	8 (27.6%)	
Insulin	4 (11.4%)	3 (10.3%)	
Hypertension in pregnancy	5 (4.3%)	8 (6.8%)	0.57^b^
Anemia in pregnancy	19 (16.2%)	24 (20.5%)	0.40
Asthma	6 (5.1%)	4 (3.4%)	0.75^b^

Data expressed as mean ± standard deviation or number (%). Analyses by Student t test for comparison of means for continuous data, and Chi-Square test for categorical datasets (Fisher exact test if > 20% of cells evaluated have cell value < 5). 2-sided analyses P < 0.05 for all variables

^a^Other ethnicities: 4 Indonesian, 1 Sri Lankan, 1 Portuguese

^b^Fisher’s exact test

Table 2 displays the primary outcome measure for spontaneous vaginal delivery and operative deliveries (cesarean and instrumental vaginal deliveries): 60/117 (51.3%) EMEP vs. 52/117 (44.4%) HB, RR 1.15 95%CI (0.88–1.51) *P* = 0.30. The cesarean to vaginal delivery (spontaneous vaginal and instrumental vaginal deliveries) comparison was 42/117 (35.9%) EMEP vs. 54/117 (46.2%) HB, RR 0.78 95%CI (0.57–1.06) *P* = 0.14. The indications for cesarean delivery were similar.

**Table 2:** Primary outcome after randomization to eye-mask and earplugs or headband

Outcomes	Eye-mask-earplugs *n* = 117	Headband *n* = 117	RR (95% CI)	NNT_b_ (95% CI)	*P* value
Mode of delivery					0.26
Spontaneous vaginal delivery	60 (51.3%)	52 (44.4%)	1.15 (0.88–1.51)^a^		0.30^1^
Instrumental vaginal delivery	15 (12.8%)	11 (9.4%)			
Cesarean delivery	42 (35.9%)	54 (46.2%)	0.78 (0.57–1.06)^b^		0.14^b^
Indications of Cesarean delivery	*n* = 42	*n* = 54			0.28
Non-reassuring fetal status	21 (50.0%)	22 (40.7%)			
Abnormal lie/presentation	1 (2.4%)	5 (9.3%)			
Poor progress of labor	11 (26.2%)	21 (38.9%)			
Failed induction of labor	5 (11.9%)	3 (5.6%)			
Others^c^	4 (9.5%)	3 (5.6%)			

Data expressed as number (%). Analyses by Chi Square test for categorical datasets. 2-sided P < 0.05 for all variables

^a^Spontaneous vaginal delivery compared to operative delivery (instrumental vaginal and Cesarean delivery)

^b^Cesarean delivery compared to vaginal delivery (spontaneous vaginal and instrumental vaginal delivery)

^c^Other indications: 3 for suspected macrosomia, 1 severe pre-eclampsia, 1 suspected cephalopelvic disproportion, 1 prolonged second stage and 1 maternal request

Table 3 lists the secondary outcomes. Based on the trial sleep outcome-related questionnaire asked at two weeks into the intervention period, participants self-reported longer night sleep duration was 7.0 ± 1.2 vs. 6.6 ± 1.5 h *P* = 0.04, agreed that they had slept better: 87/117 (74.4%) vs. 48/117 (41.0%) RR 1.81 95%CI (1.42–2.30) NNTb 4 (2.2–4.7) *P* < 0.001, expressed higher satisfaction with their allocated sleep aid: median [interquartile range] 7 [6.0–8.0] vs. 6 [5.0–7.5] *P* < 0.001 and higher self-reported use of their allocated sleep aid (a compliance measure): 5 [3.0–7.0] vs. 4 [2.0–5.0] times per week *P* = 0.002 with EMEP. The labor induction rate was not different across trial arms 50/117 (42.7%) EMEP vs. 49/117 (41.9%) HB, *p* = 1.00. However, indications for labor induction were significantly different (*p* = 0.02), especially with regard to gestational hypertension, pre-labor rupture of membranes and prolonged pregnancy. 1-min Apgar score was lower in the HB arm but the clinically important Apgar < 4 at 1 min metric [22] was not encountered in either arm. Other labor, birth and neonatal outcomes were not significantly different.

**Table 3:** Secondary outcomes after randomization to eye-mask and earplugs or headband

Outcomes	Eye-mask-earplugs *n* = 117	Headband *n* = 117	RR (95% CI)	NNT_b_ (95% CI)	*P* value
Night sleep duration (hours): self-reported	7.0 ± 1.2	6.6 ± 1.5			0.04
Participants’ satisfaction with sleep aid^a^	7 [6.0–8.0]	6 [5.0–7.5]			< 0.001
Slept better with sleep aid					
Agree^b^	87 (74.4%)	48 (41.0%)	1.81(1.42–2.30)	NNT_b_ 4 (2.2–4.7)	< 0.001
Do not agree^b^	30 (25.6%)	69 (59.0%)			
Compliance					
Sleep aid use (times per week)	5 [3.0–7.0]	4 [2.0–5.0]			0.002
Did not use sleep aid (0 times per week)	1 (0.9%)	9 (7.7%)	0.11(0.01–0.86)	NNT_b_ 15 (8.4–57.8)	0.02^ g^
**Maternal outcomes**					
Gestational age at delivery (weeks)	38.6 ± 1.2	38.6 ± 1.3			0.91
Preterm birth < 37 weeks	7 (6.0%)	9 (7.7%)	0.78(0.30–2.02)		0.80^ g^
Recruitment to delivery interval (weeks)	3.5 ± 1.4	3.5 ± 1.6			0.92
Estimated blood loss during delivery (ml)	350 [300–400]	400 [300–550]			0.25
Induction of labor	50 (42.7%)	49 (41.9%)	1.02(0.76–1.38)		1.00
Indications for induction	(*n* = 50)	(*n* = 49)			0.02
Small for gestational age	14 (28.0%)	11 (22.4%)			
Gestational diabetes	17 (34.0%)	10 (20.4%)			
Gestational hypertension	1 (2.0%)	6 (12.2%)			
Pre-labor rupture of membrane	5 (10.0%)	1 (2.0%)			
Large for gestational age	3 (6.0%)	5 (10.2%)			
Prolonged pregnancy	2 (4.0%)	11 (22.4%)			
Others^c^	8 (16.0%)	5 (10.2%)			
Methods of induction					
Amniotomy	27 (23.1%)	23 (19.7%)			0.95
Mechanical (Foley’s catheter)	42 (35.9%)	46 (39.3%)			
Prostaglandins	4 (3.4%)	3 (2.6%)			
Oxytocin	11 (9.4%)	12 (10.3%)			
Analgesia use in labor					
Epidural	31 (26.5%)	34 (29.1%)			0.85
Pethidine	8 (6.8%)	11 (9.4%)			
Entonox alone	10 (8.5%)	12 (10.3%)			
Entonox & pethidine	37 (31.6%)	31 (26.5%)			
**Neonatal outcomes**					
Birthweight (kg)	2.91 ± 0.38	2.94 ± 0.40			0.55
	*n* = 116^d^	*n* = 117			
Apgar score at 1-min Score ≥ 4	9 [9–9] 116 (100%)	9 [9–9] 117 (100%)			0.02 .^e^
Apgar score at 5-min Score ≥ 7	10 [10–10] 116 (100%)	10 [10–10] 117 (100%)			0.06 .^e^
	*n* = 107	*n* = 109			
Umbilical cord arterial blood pH	7.29[7.21–7.34]	7.31[7.25–7.34]			0.49
Neonatal admission	9 (7.7%)	11 (9.4%)			0.82
Indications for neonatal admission	(*n* = 9)	(*n* = 11)			
Infant of diabetic mother	1 (0.9%)				0.48
Transient tachypnea of newborn	5 (4.4%)	7 (6.1%)			
Presumed sepsis	2 (1.7%)	2 (1.7%)			
Low birthweight		1 (0.9%)			
Others^f^	1 (0.9%)	1 (0.9%)			

Data expressed as mean ± standard deviation, median [interquartile range] or number (%). Analyses by Student t test for continuous data, Chi Square test for categorical datasets (Fisher exact test if > 20% of cells evaluated have cell value < 5) and Mann Whitney U test for non-parametric data (assessed by Kolmogorov–Smirnov test) or ordinal data. 2-sided P < 0.05 for all variables

^a^11-point visual numerical rating score (VNRS), with 0 representing completely dissatisfied and 10 representing completely satisfied

^b^Recategorization of Likert scale responses: “agree” includes strongly or somewhat agree; “Do not agree” includes neither agree nor disagree, somewhat disagree and strongly disagree

^c^Other indications: 6 for non-reassuring fetal status, 2 for prolonged latent phase of labor, 2 for indeterminate antepartum hemorrhage, 3 for maternal medical condition

^d^One participant delivered in private center had no neonatal outcome data

^e^ No statistics are computed because the score ≥ 4 and ≥ 7 are constant

^f^ Other indications: 1 for infant of retroviral positive mother and 1 for infant with cleft lip and palate

^g^Fisher’s exact test

Post hoc for sensitivity per protocol analysis based on compliance, we performed two analyses, excluding the 10 cases where the allocated sleep aid was never used at the 2-week assessment and excluding where the sleep aid was used for only 0–3 times per week (at least 4 nights per week is equivalent ≥ 57% compliance). These analyses indicated that our intention to treat findings for spontaneous vaginal delivery, agreed to having slept better and satisfaction with sleep aid were unchanged. However, on attrition to smaller numbers, EMEP prolongation on night sleep duration was attenuated and was non-significant. Post hoc additionally, we combined the data of the trial arms to evaluate the effect of self-reported night sleep duration and qualitative agreement of having slept better with a sleep aid on spontaneous vaginal delivery and Cesarean rates. Long (≥ 7 h cut off, based on best dichotomization to top half vs. bottom half night sleep duration comparison within our entire trial sample) sleepers at 2-weeks into the trial had a higher spontaneous vaginal delivery rate 84/154 (54.5%) vs. 28/80 (35%) RR 1.56 95% CI (1.12–2.17) NNT_b_ 6 (3.1–15.5) *P* = 0.005 and a lower Cesarean delivery rate 53/154 (34.4%) vs. 43/80 (53.8%) RR 0.64 95% CI (0.48–0.86) NNT_b_ 6 (3.1–16.4) *P* = 0.004. Using the cut-off for short sleep of < 6 h our trial entry sleep duration instead, results were still significant and the magnitude point estimate marginally improved suggesting these associations were apparently consistent and robust. Participants who agreed they have slept better with the sleep aid had spontaneous higher vaginal delivery rate 72/135 (53.3%) vs. 40/99 (40.4%) RR 1.32 95% CI (0.99–1.76) *P* = 0.05 but not significantly lower Cesarean delivery rate 49/135 (36.3%) vs. 47/99 (47.5%) RR 0.77 95% CI (0.57–1.04) *P* = 0.09 (Supplementary Table S1).

There was no major harm (e.g., major incidents at night at home associated with the use of the sleep aids).

## Discussion

The primary outcome, spontaneous vaginal delivery rate although higher in the EMEP arm, the result (RR 1.15 95% CI 0.88–1.51) was not significant. The observed effect of EMEP on this outcome was smaller than that from pilot data (RR 1.2 95% CI 0.9–1.9) we used from Teo et al. [10] for sample size calculation. In our present trial the mode of delivery outcome metric that showed the highest magnitude point estimate was for the reduction in cesarean delivery (RR 0.78 95% CI 0.57–1.06) but this also did not reach significance. Compared to Teo et al. [10] who had a total trial sample size of 56 predicated on actigraphy derived night sleep duration as primary outcome, our sample size was 234 and our primary outcome was on spontaneous vaginal delivery rate. Teo et al. [10] intervention was sleep aid use for 1 week; in this trial, gestational age at recruitment was very similar to Teo et al. [10] at 35 weeks but our sleep aid use was to be continued until delivery.

EMEP demonstrated positive results over HB on self-reported night sleep hours, agreement to having slept better, satisfaction with the sleep aid. The latter two outcome findings were entirely consistent with Teo et al. [10]. However, Teo et al. [10] did not show a significant increase in night sleep duration of EMEP over sham HB with demonstrated positive placebo effect which we had done. Compliance was also higher with EMEP. We did not collect data to allow evaluation on whether participants felt that HB might be a sham resulting in attrition in its use or that the perception that EMEP use produced longer and better sleep drove adherence to its use.

In contrast to Teo et al.’s [10] finding of a significant reduction (RR 0.3 95% CI 0.1–0.8) in the labor induction rate with EMEP, our induction rate RR was 1.02 95% CI 0.76–1.38. The pattern of indications for labor induction was different across our trial arms, particularly with regard to the indications of gestational hypertension, gestational diabetes and pre-labor membrane rupture, but cell sizes contained small number of as few as one case only and this finding could be a Type 1 error of our results.

 At present the causal pathways of poor sleep leading to adverse pregnancy outcome are not fully understood. Neither is it known what the magnitude nor the period of improvement in sleep there has to be to drive gains on pregnancy, labor and delivery outcomes. Poor sleep in pregnancy is associated with gestational hypertension [8], gestational diabetes [7], prolonged labor and cesarean delivery [6]. Poor sleep pathways leading to operative delivery driven by inadequate glycemic control, worsening of hypertension and dysfunctional labor are thus plausible. EMEP increased self-reported night sleep duration and sleep quality over HB in the present trial. In a previous trial [10], HB demonstrates a significant placebo effect increasing the actigraphy derived night sleep duration; in a real-world situation of EMEP vs. a no intervention control arm, the effect of EMEP on sleep duration and potential subsequent gains on reducing operative delivery could plausibly be even greater. Within our trial population on post hoc analysis, participants who were long sleepers and those who reported better sleep after two weeks with their allocated sleep aid had significantly lower operative delivery rate. These rationales provide foundation to support EMEP as an effective sleep aid that could lower the operative delivery rate. Further study is warranted.

### Strengths and limitations

Strengths: We evaluated EMEP in the home setting in late pregnancy with a sham control to assess the “pure” above placebo effect of EMEP on an important clinical outcome of spontaneous vaginal delivery compared to operative delivery. Our primary outcome was clearly defined and easy to ascertain and we had complete data on this metric. Our sample size was based on pilot data from a smaller earlier trial from our center. Analysis was by intention to treat.

Limitations: The effect of EMEP on spontaneous vaginal delivery within our trial was smaller than that from the pilot guidance data, effectively underpowering our trial; post hoc calculation indicated that our sample size of 234 had only 18.7% power to assess spontaneous vaginal delivery rates of 51.3% vs. 44.4% that we found. We used self-reported night sleep duration; actigraphy derived sleep duration was generally preferred over self-reported duration [23] with polysomnography being the gold standard [24]. However, self-reported sleep duration was widely used in the sleep literature in pregnancy [6–9, 20, 25]. The reported compliance to sleep aid use was also generally relatively low but was significantly higher in the EMEP arm which might also reduce power but sensitivity analysis on per protocol compliance and adherence considerations did not demonstrate a major impact from these factors. We did not perform post hoc statistical correction for multiple testing of secondary outcomes hence.results concerning secondary endpoints can only have an exploratory rather than a confirmatory interpretation [26]. We did not collect the granular data on night sleep duration at recruitment beyond dichotomization to < 6 h vs ≥ 6 h.

## Conclusion

The use of eye-mask and earplugs at home in late pregnancy do not increase the spontaneous vaginal delivery rate in nulliparous women. As the sleep aid improves sleep parameters over a sham device with proven placebo effect, given its low cost, simplicity of use and well tolerated nature, further study is warranted predicated on reducing cesarean or operative delivery rates.

## Supplementary Information

**Additional file 1: Supplementary Table S1. **Post hoc sensitivityanalyses.

## Data Availability

The datasets used and/or analyzed during the current study are available from the corresponding author on reasonable request.

## Abbreviations

EMEP: Eye-masks and earplugs
HB: Headband
VNRS: Visual numerical rating scale
