# Supplementary material for: Eye-masks and earplugs compared to headband in nulliparas on increasing spontaneous vaginal delivery: a randomized trial
Source: BMC Pregnancy Childbirth. 2023 May 24;23:378. doi: 10.1186/s12884-023-05685-4 (PMC10207728; doi:10.1186/s12884-023-05685-4)
Supplement: Supplementary file 1 — Additional file 1: Supplementary Table S1. Post hoc sensitivityanalyses. [file 12884_2023_5685_MOESM1_ESM.docx]

**Supplementary Table S1:** Post hoc sensitivity analyses.

1. Per protocol analyses ever user [excluding never user with 0 time per week use of sleep aid]

| Outcomes | Eye-mask-earplugs  n = 116 | Headband  n = 108 | RR (95% CI) | NNT_b_ (95% CI) | P value |
| --- | --- | --- | --- | --- | --- |
| Mode of delivery |  |  |  |  | 0.25 |
| Spontaneous vaginal  delivery | 59 (50.9%) | 46 (42.6%) | 1.19(0.90-1.58)^1^ |  | 0.22^1^ |
| Instrumental vaginal  delivery | 15 (12.9%) | 11 (10.2%) |  |  |  |
| Caesarean delivery | 42 (36.2%) | 51 (47.2%) | 0.77(0.56-1.05)^2^ |  | 0.10^2^ |
|  |  |  |  |  |  |
| Night sleep duration (hours) | 7.0 ± 1.2 | 6.8 ± 1.2 |  |  | 0.19 |
| Participants’ satisfaction with sleep aid | 7.0 [6.0-8.0] | 6.0 [5.0-8.0] |  |  | 0.001 |
| Slept better with sleep aid |  |  |  |  |  |
| Agree | 87 (75.0%) | 47 (43.5%) | 1.72(1.36-2.19) | NNT_b_ 4 (2.3-5.2) | <0.001 |
| Do not agree | 29 (25.0%) | 61 (56.5%) |  |  |  |

1. Per protocol analyses compliant (i.e., at least 4 times per week sleep aid use ≥ 57% compliance)

| Outcomes | Eye-mask-earplugs  n = 80 | Headband  n = 59 | RR (95% CI) | NNT_b_ (95% CI) | P value |
| --- | --- | --- | --- | --- | --- |
| Mode of delivery |  |  |  |  | 0.42 |
| Spontaneous vaginal  delivery | 44 (55.0%) | 26 (44.1%) | 1.25(0.88-1.77)^1^ |  | 0.20^1^ |
| Instrumental vaginal  delivery | 10 (12.5%) | 8 (13.6%) |  |  |  |
| Caesarean delivery | 26 (32.5%) | 25 (42.4%) | 0.77(0.50-1.18)^2^ |  | 0.23^2^ |
|  |  |  |  |  |  |
| Night sleep duration (hours) | 7.1 ± 1.1 | 6.9 ± 1.1 |  |  | 0.35 |
| Participants’ satisfaction with sleep aid | 7.0 [7.0-8.0] | 7.0 [5.0-8.0] |  |  | 0.05 |
| Slept better with sleep aid |  |  |  |  |  |
| Agree | 71 (88.8%) | 34 (57.6%) | 1.54(1.22-1.94) | NNT_b_ 4(2.2-6.0) | <0.001 |
| Do not agree | 9 (11.3%) | 25 (42.4%) |  |  |  |

Data expressed as number (%). Analyses by Fisher's exact test for 2x2 categorical datasets, Chi Square test for larger than 2x2 categorical datasets. 2-sided P<0.05 for all variables.

^1^Spontaneous vaginal delivery compared to operative delivery (instrumental vaginal and Caesarean delivery).

^2^Caesarean delivery compared to vaginal delivery (spontaneous vaginal and instrumental vaginal delivery).

1. Comparison of slept better with sleep aid and mode of delivery outcomes

| Outcomes | Mode of delivery  n = 234 | | RR (95%CI) | NNT_b_ (95% CI) | P value |
| --- | --- | --- | --- | --- | --- |
|  | Spontaneous vaginal delivery | Operative delivery^1^ |  |  |  |
| Slept better with sleep aid  Agree  Do not agree | 72 (53.3%)  40 (40.4%) | 63 (46.7%)  59 (59.6%) | 1.32 (0.99-1.76) |  | 0.05 |

| Outcomes | Mode of delivery  n = 234 | | RR (95%CI) | NNT_b_ (95% CI) | P value |
| --- | --- | --- | --- | --- | --- |
|  | Cesarean delivery | Vaginal delivery^2^ |  |  |  |
| Slept better with sleep aid  Agree  Do not agree | 49 (36.3%)  47 (47.5%) | 86 (63.7%)  52 (52.5%) | 0.77 (0.57-1.04) |  | 0.09 |

1. Comparison of self-reported sleep duration and mode of delivery outcomes

| Outcomes | Mode of delivery  n = 234 | | | RR (95%CI) | NNT_b_ (95% CI) | P value |
| --- | --- | --- | --- | --- | --- | --- |
|  | Spontaneous vaginal delivery | Operative delivery^1^ | |  |  |  |
| Night sleep duration (hours) |  |  | |  |  |  |
| ≥ 7  n = 154 | 84 (54.5%) | 70 (45.5%) | | 1.56 (1.12-2.17) | NNT_b_ 6(3.1-15.5) | 0.005 |
| < 7  n = 80 | 28 (35.0%) | 52 (65.0%) | |  |  |  |
| Outcomes | Mode of delivery  n = 234 | | | RR (95%CI) | NNT_b_ (95% CI) | P value |
|  | Cesarean delivery | | Vaginal delivery^2^ |  |  |  |
| Night sleep duration (hours) |  |  | |  |  |  |
| ≥ 7  n = 154 | 53 (34.4%) | 101 (65.6%) | | 0.64 (0.48-0.86) | NNT_b_ 6 (3.1-16.4) | 0.004 |
| < 7  n = 80 | 43 (53.8%) | 37 (46.3%) | |  |  |  |

| Outcomes | Mode of delivery  n = 234 | | | RR (95%CI) | NNT_b_ (95% CI) | P value |
| --- | --- | --- | --- | --- | --- | --- |
|  | Spontaneous vaginal delivery | Operative delivery^1^ | |  |  |  |
| Night sleep duration (hours) |  |  | |  |  |  |
| ≥ 6  n = 187 | 98 (52.4%) | 89 (47.6%) | | 1.76 (1.11-2.79) | NNT_b_ 5(2.7-13.0) | 0.006 |
| < 6  n = 47 | 14 (29.8%) | 33 (70.2%) | |  |  |  |
| Outcomes | Mode of delivery  n = 234 | | | RR (95%CI) | NNT_b_ (95% CI) | P value |
|  | Cesarean delivery | | Vaginal delivery^2^ |  |  |  |
| Night sleep duration (hours) |  |  | |  |  |  |
| ≥ 6  n = 187 | 68 (36.4%) | 119 (63.6%) | | 0.61 (0.45-0.83) | NNT_b_ 5 (2.6-13.2) | 0.005 |
| < 6  n = 47 | 28 (59.6%) | 19 (40.4%) | |  |  |  |

Data expressed as number (%). Analyses by Fisher's exact test for 2x2 categorical datasets, Chi Square test for larger than 2x2 categorical datasets. 2-sided P<0.05 for all variables.

^1^Spontaneous vaginal delivery compared to operative delivery (instrumental vaginal and Caesarean delivery).

^2^Caesarean delivery compared to vaginal delivery (spontaneous vaginal and instrumental vaginal delivery)
